# Estimation of *Coregonus ussuriensis* age, growth, and maturation in China’s Amur River

**DOI:** 10.7717/peerj.12817

**Published:** 2022-02-22

**Authors:** Jilong Wang, Wei Liu, Peilun Li, Fujiang Tang, Wanqiao Lu

**Affiliations:** 1 Heilongjiang River Fisheries Research Institute, Chinese Academy of Fishery Sciences, Harbin, China; 2 Scientific Observing and Experimental Station of Fishery Resources and Environment in Heilongjiang River Basin, Ministry of Agriculture and Rural Affairs of China, Harbin, China; 3 National Agricultural Experimental Station for Fishery Resources and Environment, Fuyuan, China

**Keywords:** *Coregonus ussuriensis*, Age and growth, Maturity, Amur River

## Abstract

This study examined the age, growth, and maturation of 1,064 *Coregonus ussuriensis* individuals that were collected monthly from the middle section of the Amur River, China between 2016 and 2018. The fork length (FL) ranged from 14.9 to 49.1 cm for males and 21.5 to 58.8 cm for females, and the body weight (BW) ranged from 72.6 to 1,348.7 g for males and 107.9 to 2,854.9 g for females. The relationship of BW and FL was expressed as: BW_♂_ = 0.0324 × FL^2.708^; BW_♀_ = 0.014 × FL^2.963^. The sample ages ranged from 2 to 8 years for males and 2 to 9 years for females. We used the von Bertalanffy function based on otolith reading and observed FL data to simulate *Coregonus ussuriensis* growth, which has been suggested to be similar to that of other Salmonidae fishes. No significant difference in growth was determined between males and females. The monthly gonad somatic index (GSI) value ranged from 0.16% to 1.69% for males and from 0.73% to 16.15% for females, with a peak in November. Additionally, the reproductive staging suggested that the *Coregonus ussuriensis* spawning season was mainly in November and December. The size at maturity (FL_50%_) for males and females was 34.9 cm and 37.9 cm respectively, and the corresponding age (T_50%_) was 4.5 and 5.1, respectively. This study provides basic information for understanding the biological characteristics of *Coregonus ussuriensis* and should aid in the assessment and management of fishery resources.

## Introduction

*Coregonus ussuriensis* Berg, a salmonid, Amur whitefish that belongs to the arctic freshwater fish faunal complex, distributes in the Amur River waters, estuary of Amur River, and southern Sea of Okhotsk (Zhang, 1995; Никольский, 1960). *Coregonus ussuriensis* is iteroparous and spawns from October to January in the Amur River (Ren, 1980). *Coregonus ussuriensis* migrates to the upper reaches of the Amur River from the lower reaches or tributaries in late autumn and migrates to the lower reaches or tributaries in late spring. *Coregonus ussuriensis* is euryhaline and has a history of living in both freshwater and seawater or brackish water (Wang et al., 2019b). Additionally, *Coregonus ussuriensis* is an important economically valuable fish with high nutritional value (Wang et al., 2018). Over recent decades, *Coregonus ussuriensis* resources in China have been damaged to a certain extent. In 1998, it was listed in the China Red Data Book of Endangered Animals (Pisces) (Yue & Chen, 1998) and classified as a “vulnerable” species. Due to the improving ecological environment of the Songhua River Basin, *Coregonus ussuriensis* resources have recovered in recent years, with the catch reaching 30t/a in China, which is half as much as it was in the 1980s (Wang et al., 2019a). Wang et al. (2019c) studied the population resources of *Coregonus ussuriensis* in China and suggested the utilization of *Coregonus ussuriensis* resources was relatively sufficient and that the resource status should be monitored continuously to avoid overfishing. Overall, the fluctuation of *Coregonus ussuriensis* resources should be watched closely and relevant research and protection measures should be implemented.

Previous studies on *Coregonus ussuriensis* in China’s Amur River have explored population structure (Dong et al., 1997, 2005), biochemical genetic structure (Ma, Shi & Dong, 2003; Liang, Chang & Dong, 2004), fecundity (Li et al., 2015), and life history (Wang et al., 2019b). Other studies have looked at biological characteristics, such as average fork length (FL) and body weight (BW), which were 40.6 cm and 702.8 g, respectively (Dong et al., 2005), and individual fecundity ranged from 1.161 × 10^4^ to 5.921 × 10^4^ in the middle reaches of the Amur River (Li et al., 2015). However, few studies have systematically and comprehensively focused on the age, growth, and maturity of *Coregonus ussuriensis* in the Amur River of China.

Age, growth, and maturity are important parameters for understanding basic biological characteristics and population dynamics and providing fundamental data for fishery management (Jackson, 2004; Campana & Thorrold, 2001). This study provides a comprehensive assessment of *Coregonus ussuriensis* biological characteristics including age, growth, and reproductive maturation. This study will increase knowledge of *Coregonus ussuriensis*’s biology and help in the assessment and management of important fishery resources in order to achieve sustainable utilization.

## Materials and Methods

### Sampling

**Figure 1:**
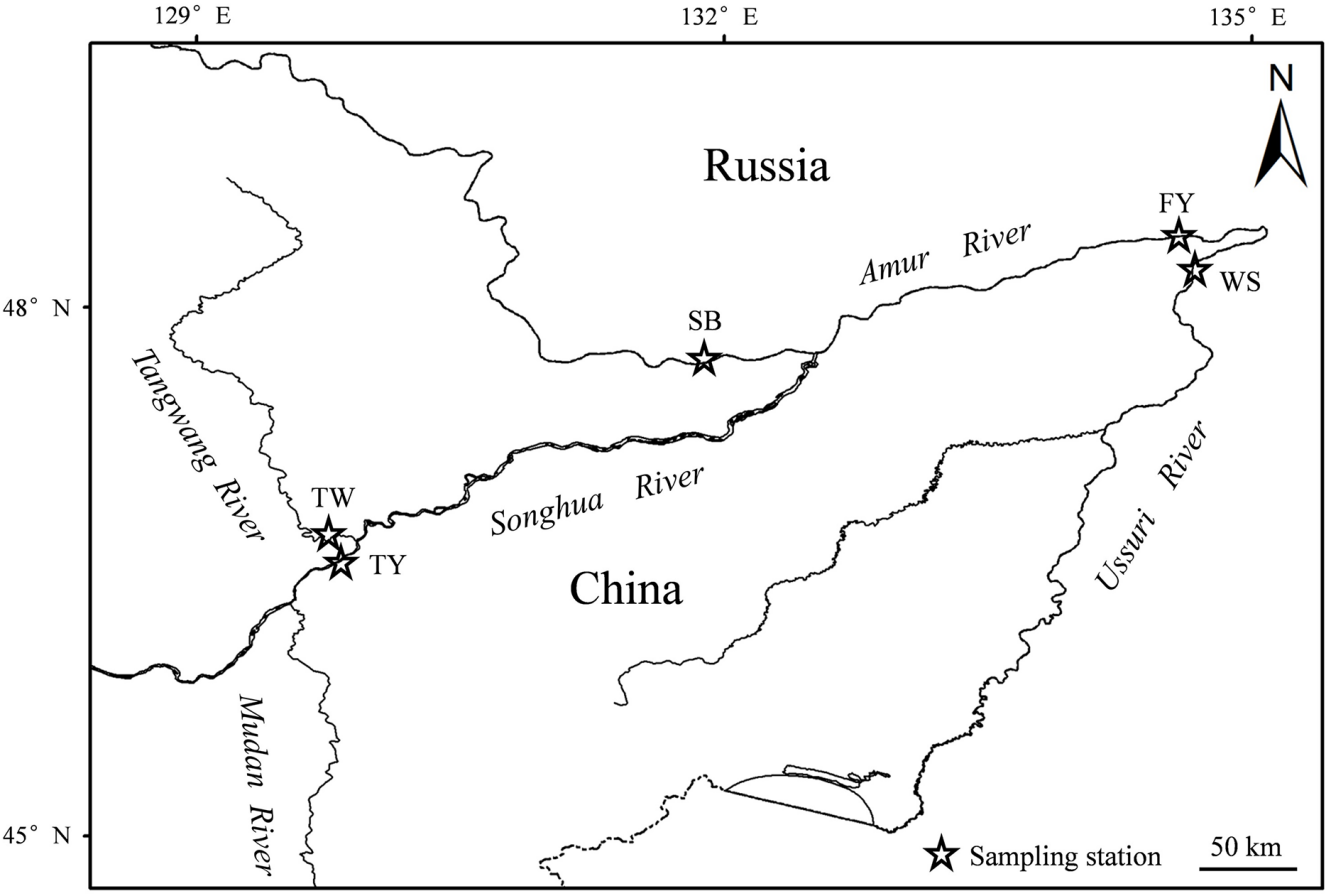
Sampling locations of *Coregonus ussuriensis* at the Suibin (SB), Fuyuan (FY) section of Amur River, Wusuzhen (WS) section of Ussuri River, and Tangyuan (TY) section of Songhua River and Tangwang River (TW). All samples were pooled for further analysis.

A total of 1,064 *Coregonus ussuriensis* samples (545 females and 519 males) were collected using floating gill nets with 4, 8 and 10 cm size mesh. The drift distance was about 1 km at the Suibin (SB) and Fuyuan (FY) sections of the Amur River, the Wusuzhen (WS) section of the Ussuri River, and the Tangyuan (TY) section of the Songhua River and Tangwang River (TW) (Fig. 1). Samples were collected monthly between July 2016 and June 2017. In order to make the number of samples exceed 30 per month, supplemental samples were collected in 2018. The samples were transported in an icebox to the laboratory, where FL, total length (TL), and BW were immediately measured to the nearest 1 mm and 0.1g, and the samples were kept as fresh as possible. Paired sagittal otoliths of each specimen were extracted, cleaned with distilled water, and stored dry in labeled tubes. There were 53, 817, 111, 31, and 52 samples of SB, FY, WS, TY, and TW, respectively, and all samples were pooled and used to analyze age, growth, and maturation. All animal experiments were conducted in accordance with the guidelines and approval of the Animal Research and Ethics Committees of Heilongjiang River Fisheries Research Institute.

### Otolith processing and aging

Each otolith was embedded in epoxy resin (Epofix, Struers, Ballerup, Denmark), mounted on a glass slide, and cut and ground to expose the core on the sagittal plane using a grinding machine equipped with a diamond cup wheel (Discoplan-TS, Struers). In order to obtain the core and most exposed annuli, the sections were further polished with 6 and 1 mm diamond paste on an automated polishing wheel (Planopol-V, Struers). The otolith microstructure of *Coregonus ussuriensis* showed characteristics of annulus that are generally represented by a sequence of alternating opaque and translucent concentric zones. The opaque zones are deposited in the rapid growth summer season, whereas the translucent zones are deposited in the slow growth period of winter (Blacker, 1974). Each otolith was read twice and if the two counts differed, the otolith was recounted until the result was consistent.

### Growth

The relationship between BW and FL was evaluated using a power function: ${\rm BW} = a{{\rm FL}^b}$. Parameters *a* and *b* were estimated using linear regression on the transformed equation: log BW = log*a* + *b*log FL. Analysis of covariance (ANCOVA) was used to detect the differences of slopes of the BW-FL relationship between sexes. Parameters *a* and *b* were regression coefficients. The von Bertalanffy growth function (VBGF) was used to fit the age and FL data (Von Bertalanffy, 1938). The formula was described as:

${L_t} = {L_\infty }(1 - {e^{ - k({t_i} - {t_0})}})$

where *L*_*t*_ is the FL at the age *t* (years), *L*_∞_ is the theoretical maximum FL, *k* is the growth coefficient, *t*_*i*_ is the age (i), *t*_0_ is the hypothetical age at FL 0. To compare and analyze the growth of *Coregonus ussuriensis* and other Salmonidae species, the growth performance index (*φ*) was used. The formula was described as: $\varphi {\rm = }{\log _{10}}k + 2{\log _{10}}{L_\infty }$.

To describe sex-specific growth with the VBGF, both male and female individuals were analyzed. An *F* test was used to compare the differences in growth between female and male samples (Chen, Jackson & Harvey, 1992).

### Reproduction

Sex was identified and maturity stage was determined using a visual evaluation according to six scales: stages I, II, III, IV, V, and VI. I: Unmature. The gonads are transparent and linear, male and female cannot be distinguished by naked eye; II: Quiescent. The gonad is still transparent, slightly enlarged, flat band, and male and female can be distinguished by naked eye. The ovaries are yellowish; III: Ripening. The weight of gonad increased. Male and female are easy to distinguish. The ovaries were light yellow and yellow, and the yolk began to deposit in the egg. The testis changes from transparent to light rose; IV: Ripeness. The weight of ovaries reached the peak and occupied most of the abdominal cavity; a large number of yolks were deposited in the ovaries, which were tightly packed into a polygonal circle. The testis turned milky white; V: Reproduction. The ovum is transparent and round, free in the ovarian cavity. The eggs and sperm can flow out of the body automatically under mild pressure; VI: Spent. The eggs and sperm have been discharged, the weight of gonad decreased significantly. The gonad membrane is loose and tough. A small number of eggs was not laid in ovary and a small amount of sperm was found in testis (Yin, 1993).

To obtain the size at maturity, changes in the proportion of mature individuals with FL were fitted to a logistic regression (described below) using the least squares method:

${p_i} = \displaystyle{1 \over {1 + {e^{ - (r \cdot {x_i} + q)}}}};$

${\rm FL}_{50\%} = -q/r$

where *p*_*i*_ is the proportion of mature individuals to FL, *q* is a constant, *r* is the intercept and FL_50%_ is the size at first maturity, which was calculated using the formula. Only individuals from the spawning period (October and November) were used for this analysis. The gonad is considered to be mature at stage IV or higher.

Gonad somatic index (GSI) is used to calculate the development of gonads in fish, and the formula is described as:

$GSI = \displaystyle{{\rm GW} \over {\rm BW}} \times 100\%$

where GW is the gonad weight. Only the samples that had gonads at stage III and higher were used to determine GSI.

## Results

### Size structure

**Figure 2:**
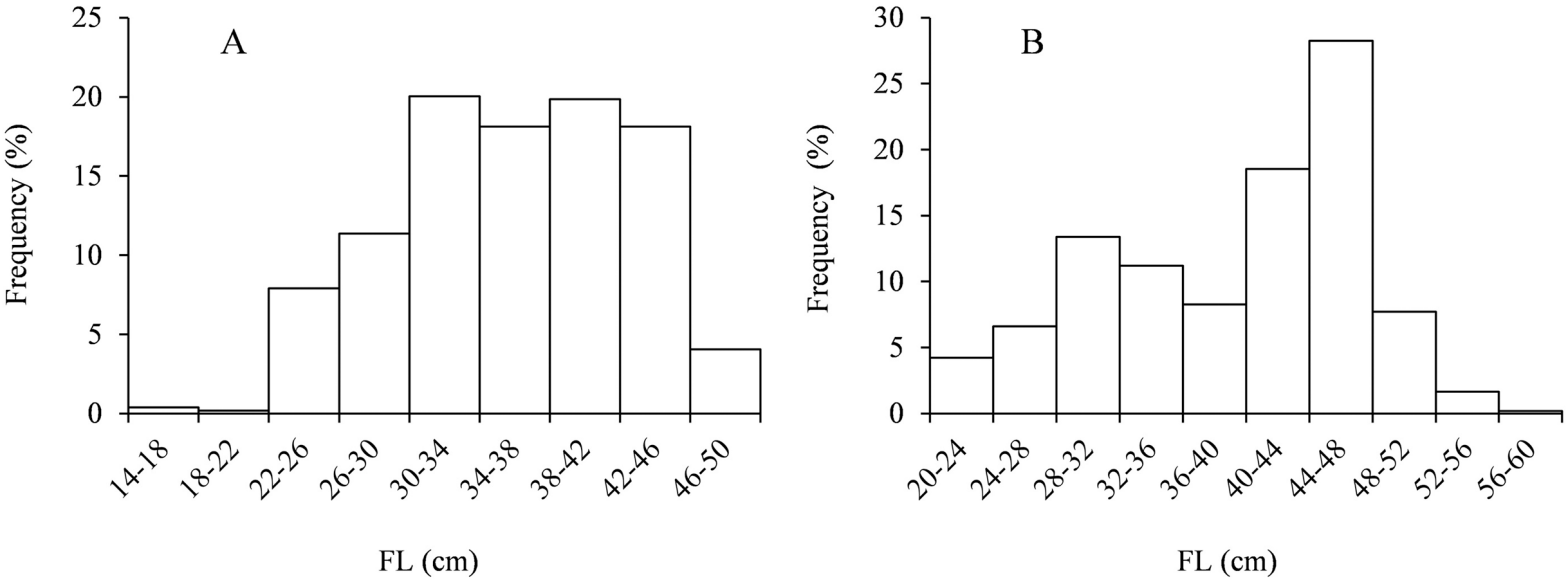
The frequency distribution of FL with 4 cm interval for (A) males and (B) females. The FL for most of the males (76.1%) ranged from 30 to 46 cm and most of females (79.6%) ranged from 28 to 48 cm.

The sampled male *Coregonus ussuriensis* had FL that ranged from 14.9 to 49.1 cm (with an average of 36.0 ± 6.6 cm) and BW that ranged from 72.6 to 1,348.7 g (with an average of 574.5 ± 263.6 g). Females had a range of 21.5 to 58.8 cm FL (with an average of 39.3 ± 8.0 cm) and 107.9 to 2,854.9 g BW (with an average of 834.7 ± 439.3 g). The FL for most of the males (76.1%) ranged from 30 to 46 cm, and most females (79.6%) ranged from 28 to 48 cm (Fig. 2). The body size of males was significantly smaller than that of females.

### Age structure

Overall, 519 males and 545 females successfully aged. The ages ranged from 2 to 8 in males, and from 2 to 9 in females (Table 1). 78.4% of males were ages 4, 5, or 6, and 82.0% of females were ages 4, 5, 6, or 7. The maximum age was 8 for males with an average FL of 47.3 cm and 9 for females with an average FL of 52.5 cm, while the minimum age was 2 for both males and females with FL of 14.9 cm and 21.5 cm, respectively.

**Table 1:** Age structure of *Coregonus ussuriensis* samples. Average FL with SD, range of FL, and sample number of different age groups are given. The age ranged from 2 to 8 for males, and 2 to 9 for females.

Age	Male			Female		
	*n*	Mean FL/cm	FL range/cm	*n*	Mean FL/cm	FL range/cm
2	5	20.58 ± 4.26	14.9–25.8	5	23.02 ± 0.87	21.5–23.9
3	65	25.85 ± 2.64	22.0–32.6	55	25.99 ± 3.06	21.5–32.5
4	139	32.07 ± 2.63	26.0–38.9	98	31.20 ± 2.59	25.5–38.6
5	153	37.44 ± 3.65	28.5–45.5	97	37.29 ± 4.46	27.3–44.4
6	115	42.23 ± 3.08	34.5–49.1	121	43.32 ± 2.59	38.5–49.5
7	40	43.92 ± 2.15	39.6–47.3	131	46.34 ± 2.21	41.2–52.9
8	2	47.3 ± 0.6	47.6–47.9	33	49.11 ± 2.82	43.7–55.5
9				5	52.54 ± 3.55	47.8–58.8

### Growth characteristics

**Figure 3:**
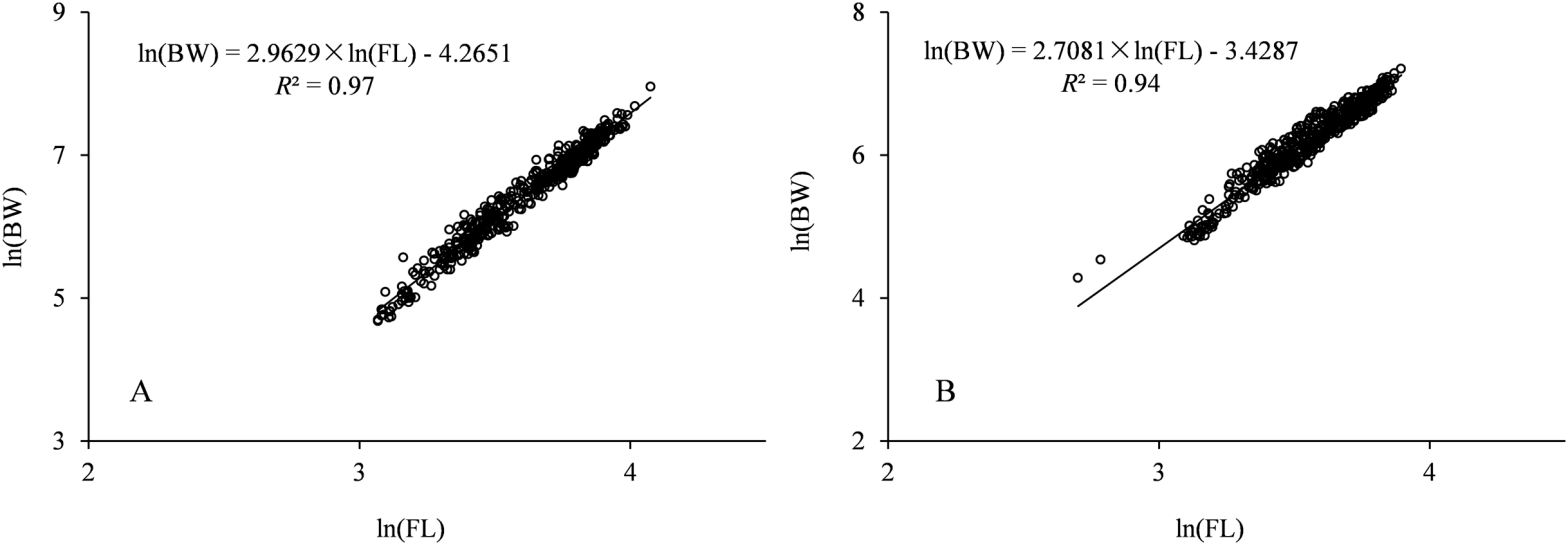
BW-FL relationship of *Coregonus ussuriensis* females (A) and males (B). Parameters *a* and *b* were estimated by linear regression for the log-transformed equation. The parameter *b* is 2.708 with 95% confidence [2.651–2.765] for males, and 2.963 with 95% confidence [2.919–3.007] for females.

The regression functions of BW and FL relationships (Fig. 3) were expressed as: BW_♂_ = 0.0324 × FL^2.708^ (*R*^2^ = 0.94, *n* = 519) and BW_♀_ = 0.014 × FL^2.963^ (*R*^2^ = 0.97, *n* = 545). For males, the parameter *b* was 2.708 with 95% confidence [2.651–2.765]; for females, the parameter *b* was 2.963 with 95% confidence [2.919–3.007]. Significant differences in the relationships between BW and FL in females and males were indicated using ANCOVA (*P* < 0.01). The allometric index value (*b*) was not significantly different from 3 (*t*-test, *t*_♂_ = 1.12, *P* > 0.05; *t*_♀_ = 0.63, *P* > 0.05) for males and females, indicating a tendency for isomeric growth in *Coregonus ussuriensis*. The FL-age relationship was described using the VBGF model, and the FL growth formulas were described as:

$\text{For males: } {\it L_t} = 59.864 \cdot (1 - {{\it e}^{ - 0.206({\it t_i} - 0.235)}});$

$\text{For females: }  {\it L_t} = 75.725 \cdot (1 - {{\it e}^{ - 0.133({\it t_i} + 0.139)}})$

**Figure 4:**
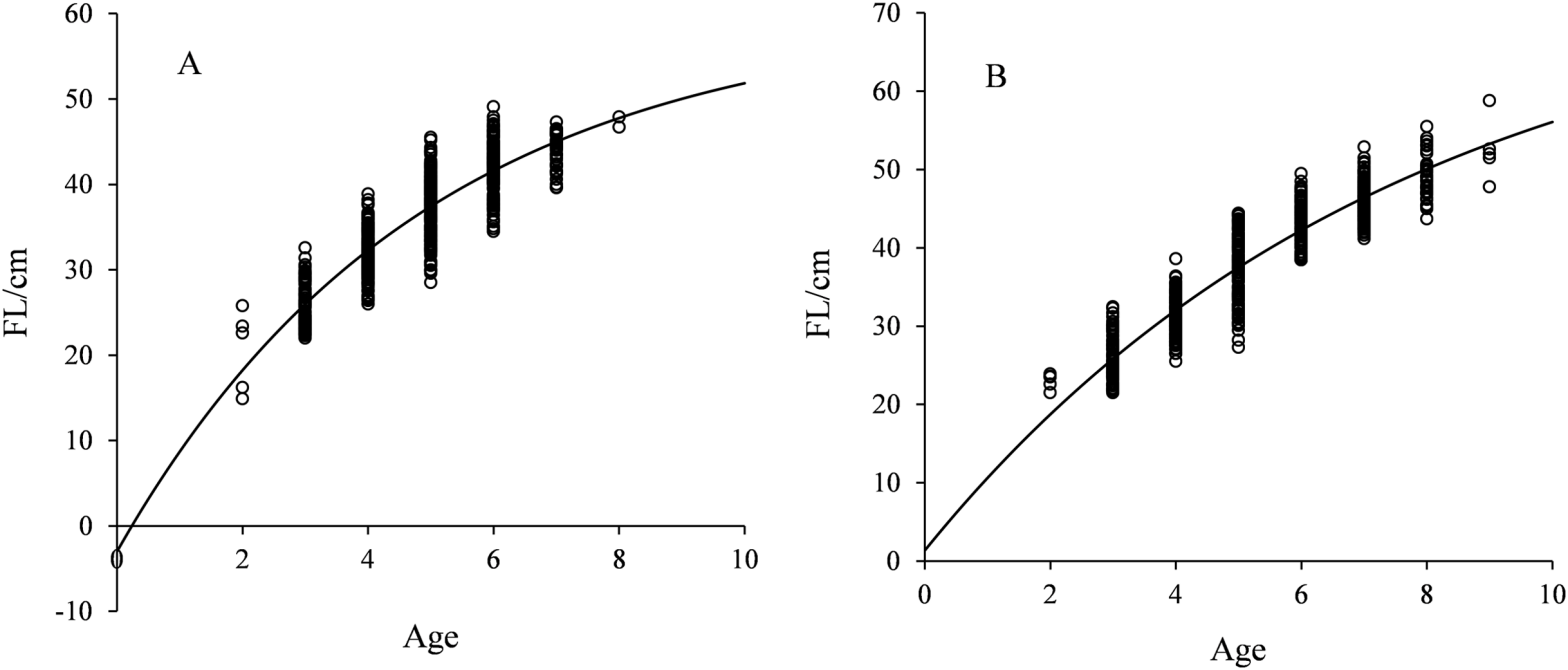
FL growth was fitted by VBGF for *Coregonus ussuriensis* males (A) and females (B). The parameters *L_∞_*, *k*, and *t*_0_ were 59.864, 0.206, and 0.235 for males, and 75.725, 0.133, and −0.139 for females, respectively. *F* test showed there was no significant difference between sexes (*F* = 0.481 < *F*_(0.05,11,14)_).

The carve of FL growth fitted by VBGF is shown in Fig. 4. *F* test showed no significant difference between sexes (*F* = 0.481< *F*_(0.05,11,14)_). The FL growth equation across all specimens (females and males) was described as:

${L_t} = 73.282 \cdot (1 - {e^{ - 0.139({t_i} + 0.153)}});$

The parameters *L*_*∞*_, *k*, *t*_0_ were 73.282, 0.139, and −0.153, with the standard error 4.580, 0.018, and 0.185 and 95% confidence [64.295–82.271], [0.104–0.174], and [−0.516 to 0.210] respectively, and the growth performance index (*φ*) was 2.87 for all the samples.

### Maturation

**Figure 5:**
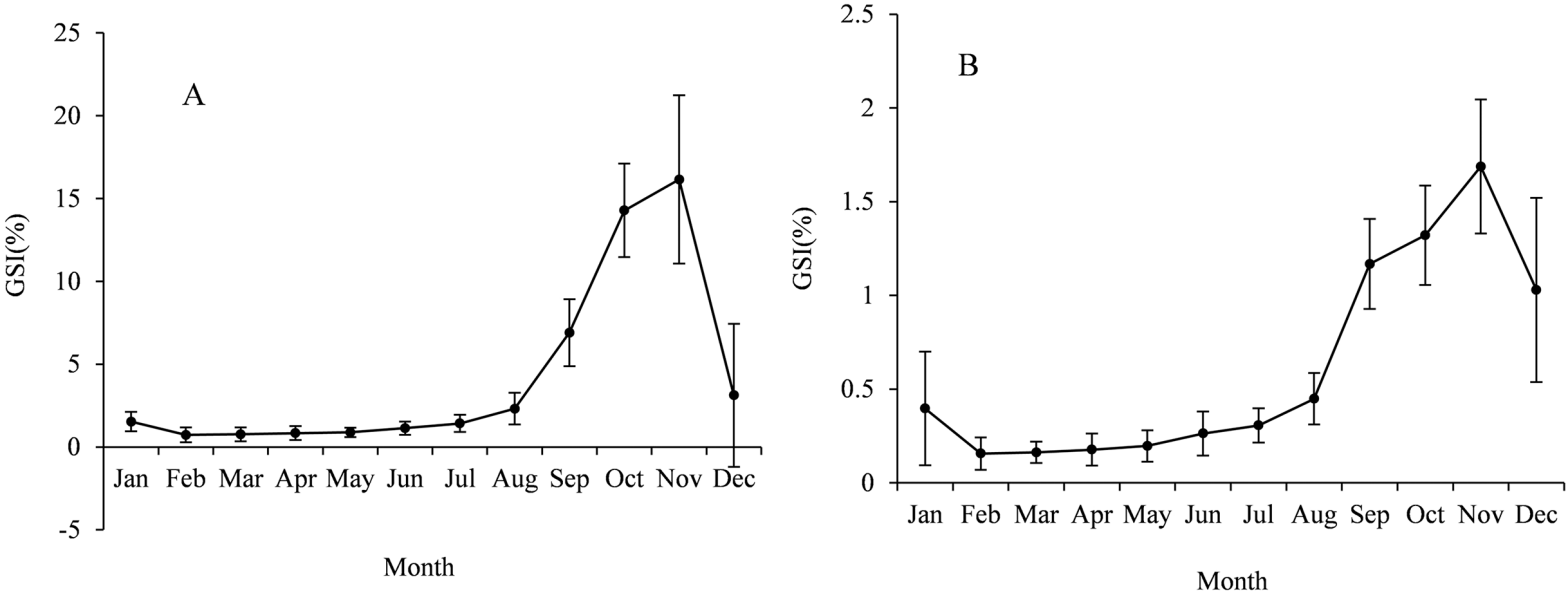
The average GSI values with standard deviation (SD) for females (A) and males (B) *Coregonus ussuriensis* samples were calculated monthly. The GSI values ranged from 0.16% to 1.69% for males, and 0.73% to 16.15% for females. The value peaked in November for both females and males.

The sex ratio (F:M) of *Coregonus ussuriensis* samples in this study was 1.05:1. We calculated the GSI for males and females monthly and the value ranged from 0.16% to 1.69% for males and 0.73% to 16.15% for females (Fig. 5). According to the results, the gonads of males and females developed rapidly from September to November and the GSI value peaked in November. The female gonads in November were mainly at stage IV and V (only 7% of the samples were at stage VI), while the samples in December were mainly at stage VI (about 90%). This indicated that the *Coregonus ussuriensis* spawning period was mainly in November and December. The monthly change trend of male GSI was similar to that of females. The smallest FL of mature samples was 29.9 cm and 36.5 cm for males and females, respectively. In the formula of the proportion of mature individuals with FL, the parameters *q* and *r* were −18.477 and 0.530, respectively, with the standard error 1.409 and 0.040 and 95% confidence [−21.808 to −15.146], [0.434–0.625] for males, and −21.289 and 0.562 with the standard error 2.163 and 0.057 and 95% confidence [−26.277 to −16.30], [0.430–0.693] for females. The size at first maturity (FL_50%_) for females and males is described using the following formulas:

$\text{For males : } {\it p_i} = \displaystyle{1 \over {1 + {{\it e}^{ - 0.530{\it x_i} + 18.477}}}};$

$\text{For females : } {\it p_i} = \displaystyle{1 \over {1 + {{\it e}^{ - 0.562{\it x_i} + 21.289}}}}$

**Figure 6:**
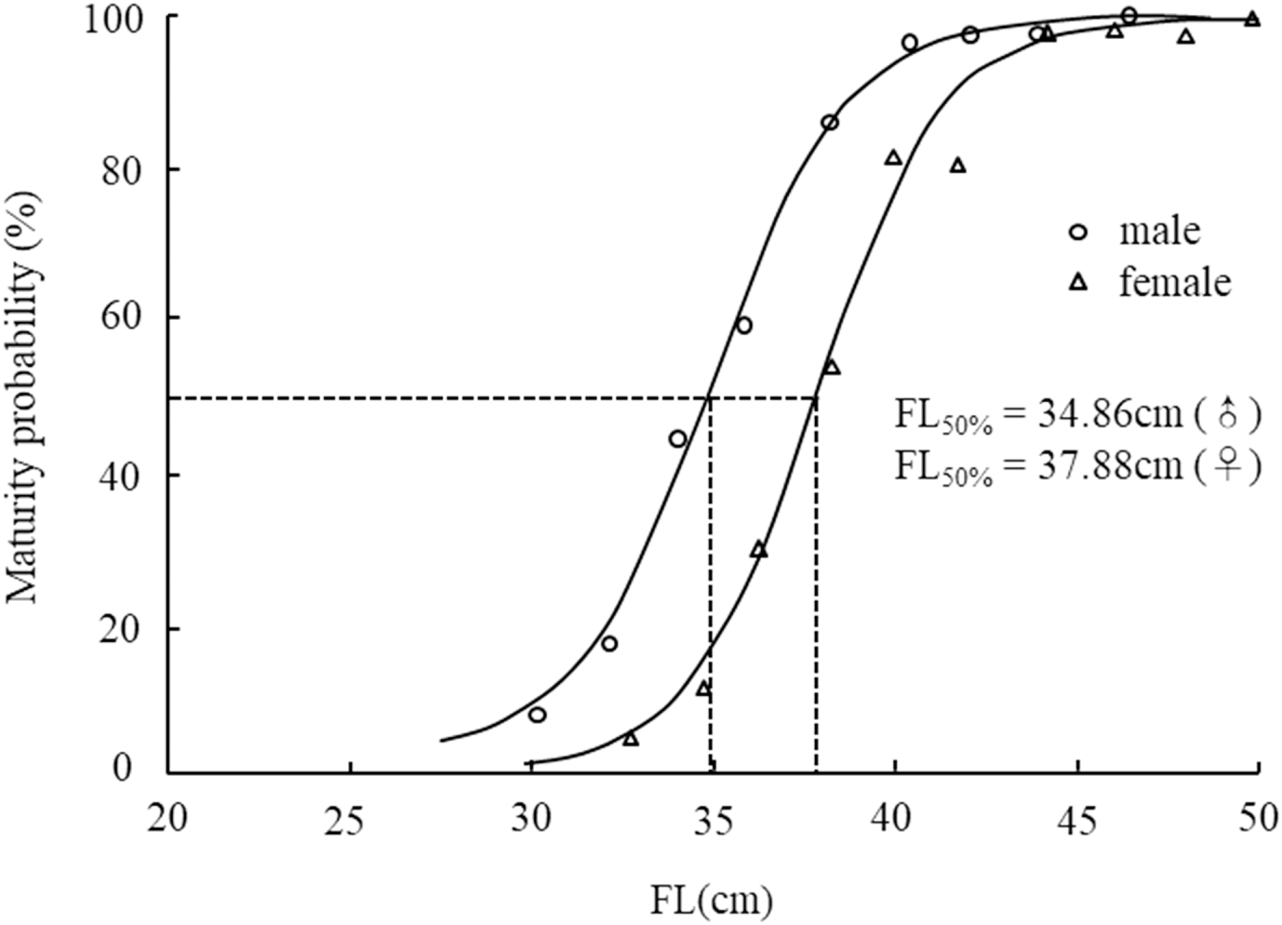
Logistic functions fitted to percent mature proportion intervals for *Coregonus ussuriensis* female and male individuals. The parameters *q* and *r* were -18.477 and 0.530 with the standard error 1.409 and 0.040 and 95% confidence [−21.808 to −15.146], [0.434–0.625] for males, and −21.289 and 0.562 with the standard error 2.163 and 0.057 and 95% confidence [−26.277 to −16.30], [0.430–0.693] for females. The FL_50%_ values for males and females were 34.9 cm and 37.9 cm, respectively. *F* test showed there was significant difference of FL_50%_ between the sexes (*F* = 063.908 > *F*_(0.01,15,17)_).

The FL_50%_ for males and females was 34.9 cm and 37.9 cm, respectively (Fig. 6). *F* test showed the significant difference of FL_50%_ between the sexes (*F* = 63.908 > *F*_(0.01,15,17)_). According to the FL growth formula, the age at first maturity (T_50%_) for males and females was 4.5 years and 5.1 years, respectively. Therefore, we concluded that males reach first sexual maturity at a smaller size or earlier than females.

## Discussion

### Body size of the samples

*Coregonus ussuriensis* is a seasonal migratory species that migrates to the Amur River, Ussuri River and Songhua River in autumn and winter. In this study, samples were collected monthly and the majority of samples were collected in autumn and winter. A large number of reproductive populations were included in the samples and the dominant FL groups were 30–46 cm and 28–48 cm for males and females, respectively. Across all of the samples, the minimum FL and BW were 14.9 cm and 72.6 g for males, and 21.5 cm and 107.9 g for females. The smaller juvenile fish (0–1 year) were not caught during this study, possibly due to the larvae migrating downstream or to the estuary of the Amur River that is nearly 1,000 km away from the FY sampling station. It did not migrate to the Amur River waters of China until the body size reached a certain size specification. Females had a larger body size (average FL 39.3 ± 8.0 cm) than males (average FL 36.0 ± 6.6 cm), which has also been observed in other Salmonidae species such as *Oncorhynchus keta* (Wang, Liu & Tang, 2013). The BW-FL relationship was described using a power exponent function where the regression coefficient *b* was not significantly different from 3, which demonstrated that both *Coregonus ussuriensis* females and males showed isomeric growth and were suitable to be simulated by VBGF. Similar conclusions were found for *Myctophum asperum* (Wang et al., 2019c) and *Electrona antarctica* (Li & Zhu, 2015). Fish length–weight relationships can be affected by many factors including season, habit, gonad maturity, sex, and stomach fullness (Tesch, 1971; Froese, 2006). The BW-FL relationships of *Coregonus ussuriensis* females and males were significantly different from each other, which was consistent with the study by Dong et al. (1997), and are related to gonad weight between the sexes. According to our results, the average *Coregonus ussuriensis* body size was smaller than the size found by a survey in 2001 (Dong et al., 2005). The size of *Coregonus ussuriensis* has shown a decreasing trend in recent years, which was also observed in *Oncorhynchus keta* from the same area in 2012 (Wang, Liu & Tang, 2013). This decreasing trend in body size should be the focus of long-term monitoring.

### Age and growth

Due to this study’s long survey time and large sample size, the number of age groups in the samples was much greater than that of previous surveys (Dong et al., 1997; Dong et al., 2005). In particular, we collected juvenile fish samples with a minimum age of 2 years for both males and females, which had never been reported in China before. However, the maximum age was 8 and 9 years for males and females, respectively, which was younger than the maximum age (10 years) found in 1994 (Dong et al., 1997). Similar to previous studies of other Salmonidae fishes (Wang, Tang & Liu, 2012; Yin et al., 2003; Xue et al., 2013), a von Bertalanffy equation was used to fit the FL growth of *Coregonus ussuriensis*, and no significant difference in FL growth was found between the sexes, although the average size of females was larger than that of males. The growth performance index (*φ*) was used to compare the growth rate among several Salmonidae species (Table 2). *Oncorhynchus keta* (Wang, Tang & Liu, 2012), *Hucho taimen* (Yin et al., 2003), and *Salmo trutta fario* (Hao et al., 2007) were shown to grow faster than *Coregonus ussuriensis*, while *Brachymystax lenok* and *S. malma* grew slower, revealing that *Coregonus ussuriensis*’s growth rate was basically in the middle of the Salmonidae fishes listed above. Generally, salmonids are cold water fishes that grow slowly. Some salmonids are popular because of their high nutritional value, delicious taste, and important commercial value. In China, several salmonids (except *Oncorhynchus keta*) have been developed for commercial breeding. The study of the growth of *Coregonus ussuriensis* will not only be helpful in the understanding of its ecological characteristics, but also in the assessment of the value of its commercial development. *Coregonus ussuriensis* is a slow-growing fish species, and the population would be difficult to restore in the instances of habitat destruction or overfishing (Musick, 1999). It is therefore essential for its sustainability to protect its habitat environment and establish effective management strategies focused on optimizing fishing size and prohibiting fishing times and areas.

**Table 2:** Growth parameters of several Salmonidae fishes. *L*_∞_, *k*, and *t*_0_ were parameters in VBGF. The growth performance index *φ* was used to compare the growth across the species, and the results showed that the growth of *Coregonus ussuriensis* was in the middle.

Species	*L*_∞_	*k*	*t*_0_	The growth performance index/φ	Survey year	Sources
*Brachymystax lenok tsinlingensis*	729.38	0.08	−0.5	4.629	2009, 2012	Xue et al. (2013)
*Oncorhynchus keta* Walbaum	900.4	0.3	−0.27	5.386	2010–2011	Wang, Tang & Liu (2012)
*Hucho taimen*	2464.1	0.0407	0.4625	5.393	2000	Yin et al. (2003)
*S. malma*	496.4	0.1825	−0.002138	4.653	1994–1996	Huang et al. (1999)
*Salmo trutta fario* L (females)	709.44	4.1656	4.0845	6.322	1999–2002	Hao et al. (2007)
*Salmo trutta fario* L (males)	797.82	0.1428	0.0058	4.959	1999–2002	Hao et al. (2007)
*Coregonus ussuriensis*	732.82	0.139	−0.153	**4.873**	2016–2018	This study

### Maturation

The monthly variation curve of GSI was unimodal and the value peaked in November, which revealed *Coregonus ussuriensis* is a determinate spawner (Fig. 5). Based on the monthly results of the gonad development stages, *Coregonus ussuriensis* mainly spawn in November and December, which is just at the beginning of icebound season, and is consistent with the results recorded by Zhang (1995). According to the results of this survey, a high proportion of mature individuals (gonads at stage IV and V) were observed in the FY, SB and WS stations in November, which suggested that the *Coregonus ussuriensis* spawning grounds were near these three sampling stations. The GSI value for female *Coregonus ussuriensis* during the spawning season (November) reached 16.15%, which is smaller than the average GSI values ranging from 18.63% to 26.94% in different age groups found by Li et al. (2015). This difference is because Li’s study used mature individuals with gonads at stage IV and V to analyze the GSI. The minimum size and age were 36.5 cm and 4 years for mature females, and 29.9 cm and 4 years for mature males, respectively, which is the same as the results found by Li et al. (2015), but smaller than those reported by Dong et al. (1997) (38 cm and 6 years). The sample size in this study is the largest of the related studies, which may have resulted in this difference of size observed at maturity. FL_50%_ is an important parameter used to indicate maturity, and this study was the first to use it for *Coregonus ussuriensis*. The FL_50%_ of males and females was 34.9 cm and 37.9 cm, respectively, and the corresponding age (T_50%_) was 4.5 years and 5.1 years, respectively, which suggested that females mature at a larger size or later than males. Previous studies found that size at maturity was influenced by genetic origin and the habitat’s physical and biotic environment (Keyl et al., 2008). Additionally, anthropogenic disturbance can also affect the FL_50%_ value, which changes to compensate with fishing pressure (Kirkpatrick, 1993; Witting, 2002). For the sustainable utilization of *Coregonus ussuriensis*, the fishing standard size should be larger than the FL_50%_ value, which should be continuously monitored as a conventional indicator in order to understand its biological characteristics and resource status. The quantitative management of the minimum fishing size of *Coregonus ussuriensis* should be more scientific and effective than the current fishery management measures, which only include the minimum mesh size and fishing gear restrictions.

## Conclusion

In this study, 1,064 *Coregonus ussuriensis* samples were collected monthly. The von Bertalanffy growth functions for all samples was described as: ${L_t} = 73.282 \cdot (1 - {e^{ - 0.139({t_i} + 0.153)}})$. The monthly GSI values ranged from 0.16% to 1.69% and 0.73% to 16.15% for males and females, respectively. *Coregonus ussuriensis* is a determinate spawner that mainly spawns in November and December. This study’s results will improve our knowledge of *Coregonus ussuriensis*’s biology and should be used to assess and manage this important resource.

## Supplemental Information

10.7717/peerj.12817/supp-1 Supplemental Information 1 FL, TL, BW and sex data.

## Additional Information and Declarations

### Data Availability

The following information was supplied regarding data availability: The basic data for the body size, age and other factors are available as a Supplemental File.
